# RETRACTION: Effect of Closed Incision Negative Pressure Wound Treatment in Vascular Surgery: A Meta‐Analysis

**DOI:** 10.1111/iwj.70260

**Published:** 2025-02-25

## Abstract

**RETRACTION:** MengX. , XieX. , LiuY. , HuangC. , WangL. , FangX. , and ChenX. , “Effect of Closed Incision Negative Pressure Wound Treatment in Vascular Surgery: A Meta‐Analysis,” International Wound Journal 21, no. 1 (2024): e14392, 10.1111/iwj.14392.37722871 PMC10788581

The above article, published online on 18 September 2023, in Wiley Online Library (http://onlinelibrary.wiley.com/), has been retracted by agreement between the journal Editor in Chief, Professor Keith Harding; and John Wiley & Sons Ltd. Following an investigation by the publisher, all parties have concluded that this article was accepted solely on the basis of a compromised peer review process. The editors have therefore decided to retract the article. The authors did not respond to our notice regarding the retraction.

**RETRACTION:** X. Meng , X. Xie , Y. Liu , C. Huang , L. Wang , X. Fang , and X. Chen , “Effect of Closed Incision Negative Pressure Wound Treatment in Vascular Surgery: A Meta‐Analysis,” International Wound Journal 21, no. 1 (2024): e14392, 10.1111/iwj.14392.37722871 PMC10788581

The above article, published online on 18 September 2023, in Wiley Online Library (http://onlinelibrary.wiley.com/), has been retracted by agreement between the journal Editor in Chief, Professor Keith Harding; and John Wiley & Sons Ltd. Following an investigation by the publisher, all parties have concluded that this article was accepted solely on the basis of a compromised peer review process. The editors have therefore decided to retract the article. The authors did not respond to our notice regarding the retraction.

